# Scientific and Technological Developments in Brain-Computer Interfaces: Dual Bibliometric Analysis of Articles and Patents

**DOI:** 10.2196/95902

**Published:** 2026-09-10

**Authors:** Junhui Wang, Yakun Yuan, Hang Xu, Guannan Luan

**Affiliations:** 1 Institute of Medical Information, Chinese Academy of Medical Sciences & Peking Union Medical College, 3 Yabao Road, Chaoyang District, Beijing, 100020, China, 1 861-812-0801

**Keywords:** Bibliometric, brain–computer interface, topic analysis, category normalized citation impact, disruptive index

## Abstract

**Background:**

Brain-computer interface (BCI) technology is undergoing rapid translation from laboratory research to clinical applications, heralding a fundamental restructuring of human-machine relationships, with profound societal implications. Existing bibliometric analyses of this domain have exclusively relied on scholarly article databases, critically overlooking patent data that is essential for capturing the full spectrum of technological innovation in this highly translational field.

**Objective:**

This study aims to investigate the overall scientific and technological trajectories, key research drivers (journals, institutions, countries, and funding agencies), as well as research topics and trends in the BCI field through a comprehensive analysis of scientific and technical literature (articles and patents).

**Methods:**

BCI-related articles (11,346) and granted patents (1551) published from 2015 to 2025 were retrieved from the Web of Science (WOS) and the incoPat database, respectively. VOSviewer, CiteSpace (Dr Chaomei Chen), Microsoft Excel, and InCites (Clarivate) were used to summarize bibliometric features. Additionally, the Disruptive Index was calculated to characterize the developmental trajectory of scientific and technological advancements in BCIs.

**Results:**

The number of BCI articles and patents has been continuously increasing over the past decade. The *Journal of Neural Engineering* published the largest number of BCI articles. The Chinese Academy of Sciences ranked first in article output, while the University of California System achieved the greatest citation impact, and Tianjin University from China led in patent filings. At the country level, China dominated article output and patent filings, whereas the United States attained the highest article citation impact and the most extensive international patent portfolios. The National Natural Science Foundation of China (NSFC) was the most prolific funding agency for BCI articles. Disruptive Index analysis revealed that BCI scientific research (article-based) maintained sustained growth in disruptiveness, whereas technological development (patent-based) demonstrated a pattern of fluctuation rather than consistent growth during the observation period. A total of 5 major research topics were identified, with neural interfaces and motor control attracting the greatest attention. In parallel, the leading technology categories were computer input or output interface devices (IPC: G06F3) and diagnostic measurement and human identification (IPC: A61B5). Citation analysis revealed an average knowledge transfer lag of 8.8 years, alongside limited bidirectional article-patent linkages.

**Conclusions:**

This study reveals a rapidly expanding yet strategically differentiated global BCI landscape dominated by China and the United States, with the former leading in output volume and the latter achieving the highest citation impact and international patent portfolios. BCI scientific research maintains active disruptive potential, whereas technological development lacks a commensurate upward trajectory; this asynchrony, compounded by prolonged knowledge transfer lag and weak article-patent linkages, points to translational challenges that require strengthened mechanisms for converting scientific discovery into technical innovations.

## Introduction

Brain-computer interface (BCI) has undergone substantial development over recent decades, establishing itself as a multidisciplinary domain spanning neuroscience, engineering, computer science, and rehabilitation medicine. Pioneering experiments, exemplified by Delgado’s remote brain stimulation via a “stimoceiver” [1], contributed to the conceptual origins of modern BCI research. BCI technology initially developed as a tool for elucidating neural mechanisms, subsequently enabling direct communication between the brain and external devices by translating neural activity into commands for controlling computers, prosthetic limbs, or other assistive systems, and later advancing to bidirectional interfaces that both record neural signals and deliver sensory feedback, enabling closed-loop systems capable of restoring motor function in paralyzed individuals through bypassing damaged neural pathways [2].

The field is currently experiencing extensive integration of AI, including deep learning and neural networks, to enhance BCI performance and customize systems to individual users [3,4]. These advances are driving the technology’s evolution from basic scientific experiments to versatile applications spanning medical rehabilitation, assistive devices, and entertainment. Continued advances in AI, coupled with sustained interdisciplinary collaboration, are anticipated to enhance both the functionality and accessibility of BCI systems.

Bibliometric analysis, as a mature quantitative methodology, enables systematic investigation of research participants, thematic evolution, and developmental trajectories within specific fields [5,6], and has been increasingly applied to the BCI field [7-15]. A state-of-the-art literature review reveals that existing bibliometric studies of this domain have predominantly focused on specific applications or subfields. Examples include disease-specific investigations such as stroke [7,8], spinal cord injury [9], and Alzheimer disease [10]; signal processing approaches including electroencephalography [11], motor imagery, and steady-state visually evoked potentials [12]; and specialized subfields such as BCI software platforms [13], emotion research [14], and brain-computer music interfaces [15].

A limited number of studies have examined the overall development of the BCI field using bibliometric methods. Hu et al [16] analyzed 100 highly cited BCI papers published from 2006‐2015, while Yin et al [2] provided a comprehensive overview of BCI research from 1990 to 2020. However, all aforementioned studies relied exclusively on article data from the Web of Science (WOS) Core Collection, PubMed, or Scopus, with no incorporation of patent data. Given the pronounced technological orientation of the BCI field, patent analysis constitutes an essential component for understanding its comprehensive development trajectory. To address this gap, this study adopts a dual-source approach integrating both articles and patents to provide a holistic characterization of scientific and technological development in the BCI domain.

## Methods

### Data Source and Search Strategy

**Figure 1.**
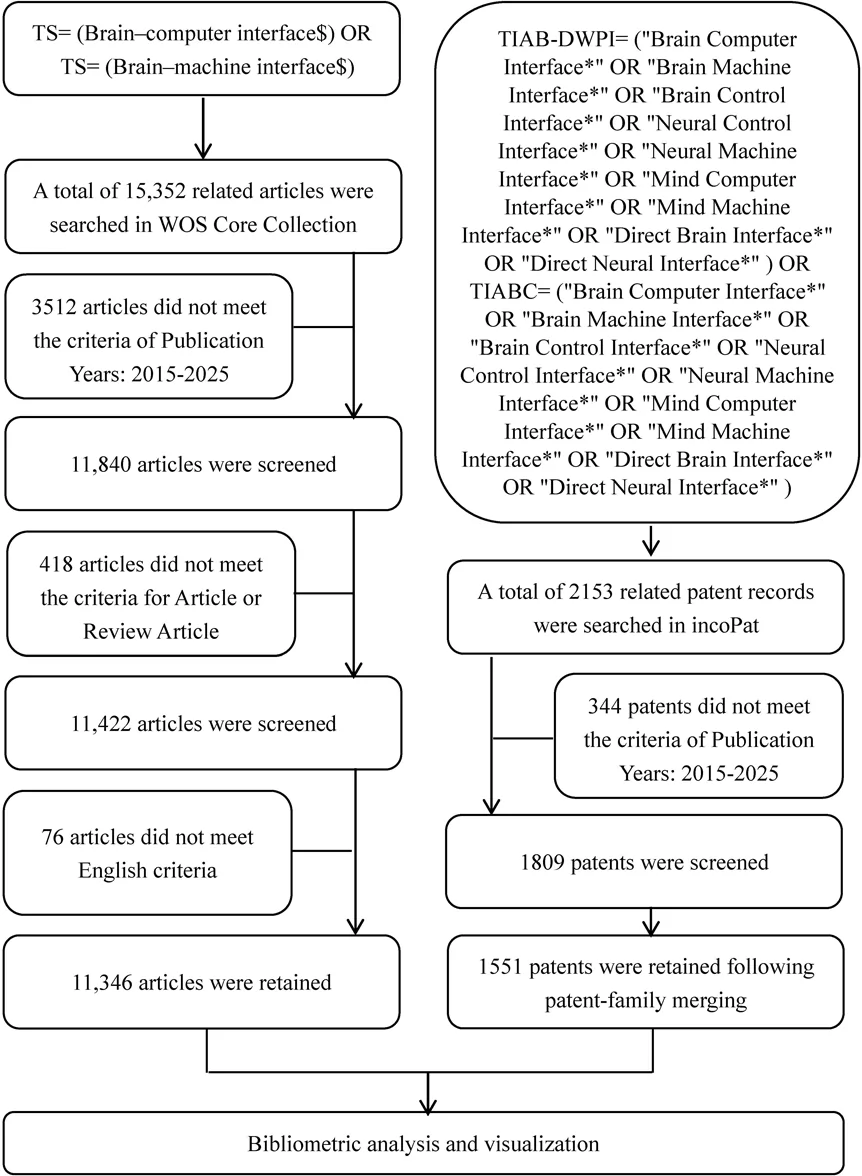
Flowchart of articles and patents screening.

This study uses both articles and patents in the field of BCI as complementary analytical data sources, with the publication timeframe restricted to 2015-2025. Specifically, article data were retrieved from the WOS Core Collection database, with the search executed on February 1, 2026, yielding a total of 11,346 BCI-related articles. Patent data were exclusively sourced from the incoPat database (Clarivate) and limited to granted patents to ensure the focus on legally protected innovations [17]; the search was conducted on June 1, 2026, retrieving 1809 patents that were later reduced to 1551 records via patent-family merging (grouping documents from identical simple patent families). The search strategies for both articles and patents are detailed in Figure 1.

### Bibliometric Analysis

Bibliometric mapping and visualization were conducted using VOSviewer (version 1.6.20; Leiden University) and CiteSpace (version 6.3.1; Dr Chaomei Chen). VOSviewer generated keyword cooccurrence networks with color-coded clusters, where internode distances indicate thematic relatedness, sizes reflect occurrence frequency, and density represents connection strength—enabling identification of research trends and hotspots. CiteSpace detected burst keywords as indicators of cutting-edge topics, visualized along a horizontal blue timeline with red segments denoting the start year, end year, and duration of each keyword [18]. Additionally, relevant descriptive analysis charts were created using Microsoft Excel (version 2021) and InCites (Clarivate).

Bidirectional citation analysis was employed to explore the translational dynamics from scientific research to technological innovation. On one hand, “backward citation analysis” was conducted at the patent level to identify granted patents that cite scientific articles, with simultaneous analysis of the time lag between patent grant years and the publication years of the cited papers. On the other hand, “forward citation analysis” was conducted at the article level to identify highly cited papers (HCPs; top 1% by citations for their category) that receive patent citations. To effectively capture the citation relationships between patents and articles, the Lens database was used as a supplementary source [19], which provides comprehensive cross-reference information for both articles and patents, in addition to the incoPat database.

### Disruptive Index

The Disruptive Index, first proposed by Funk and Smith [20], has emerged as a crucial bibliometric metric for evaluating the development trend of science and technology. This innovative metric helps researchers, policymakers, and institutions to assess how groundbreaking new studies change the direction of science and technology. The core logic of the Disruption Index is to characterize the consolidating or disruptive nature of science and technology by analyzing the changes in the citation network of articles and patents. The Disruptive Index of a focal work (article or patent) is calculated as:


$$D=\frac{n_{i}-n_{j}}{n_{i}+n_{j}+n_{k}}$$

Where *n_i_* is the number of subsequent works that cite the focal work, *n_j_* is the number of subsequent works that cite both the focal work and its references, and *n_k_* is the number of subsequent works that only cite the focal work’s references. To ensure comparability with prior research, this study used the Five-Year Disruptive Index (CD5) proposed by Park et al [21], restricting citation counts to the five-year window following each focal work. Consequently, CD5 values were calculated only for publications up to 2020, ensuring complete observation periods. To assess the current state and trajectory of scientific and technological development in the field of BCI, the CD5 was computed separately for articles and patents.

## Results

### Annual Publication Trends

**Figure 2.**
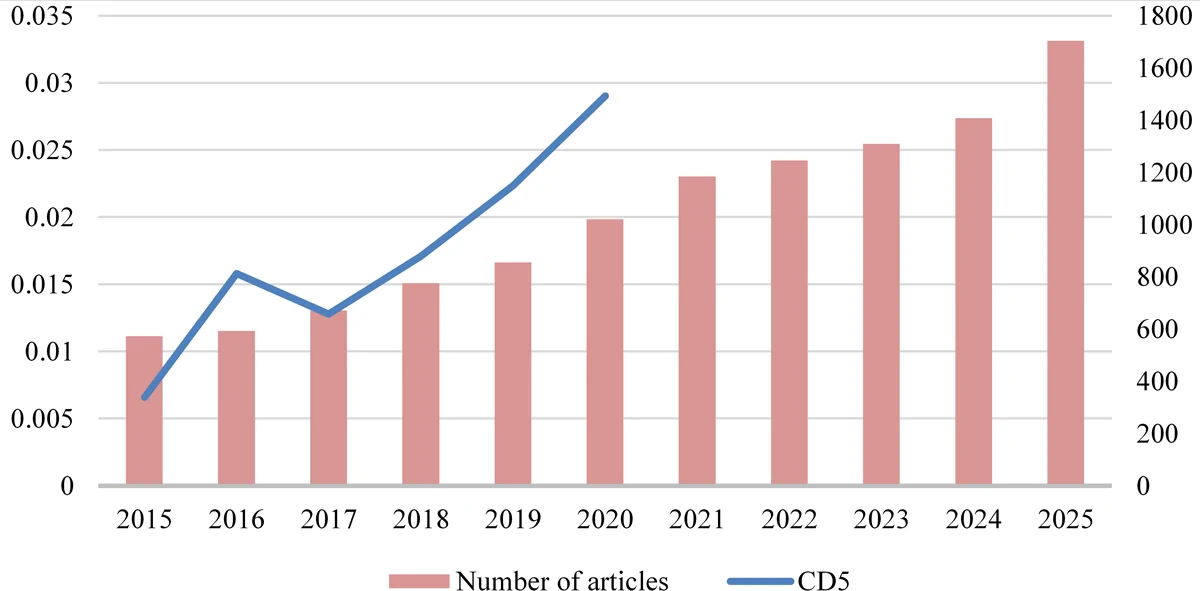
Trends of the number and CD5 of BCI articles. CD5: five-year Disruptive Index.

**Figure 3.**
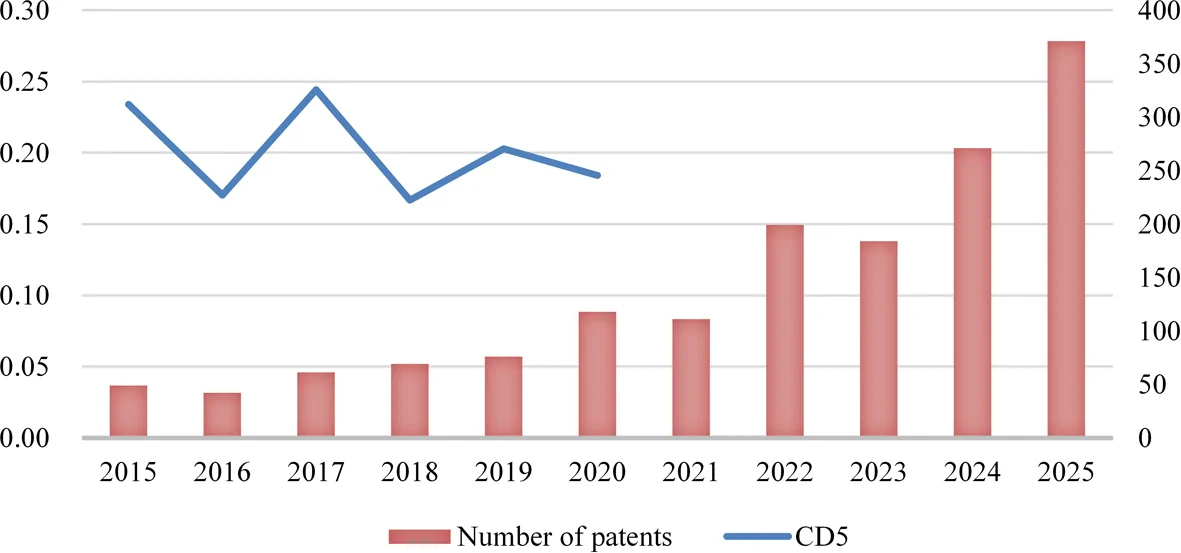
Trends of the number and CD5 of brain-computer interface (BCI) patents. CD5: Five-Year Disruptive Index.

The annual trends in article and patent outputs in the BCI field are illustrated in Figures 2 and 3. Both datasets demonstrate a sustained growth trajectory over the past decade, reaching their respective peaks in 2025 (1705 articles and 371 patents). The volume of both publication types is projected to continue increasing in 2026.

Granted BCI patents increased from 49 in 2015 to over 100 annually from 2020 onward, with notable accelerations during 2021‐2022 and 2024‐2025. These surges potentially reflect the influence of supportive policies and strategic plans worldwide, exemplified by China’s 13th Five-Year Plan for brain science (2016) and the US BRAIN Initiative 2.0 (2019), which may have propelled research and development efforts in BCI technology.

### Journal and Citation Analysis

Multimedia Appendix 1 presents the top 10 journals with the highest publication output in the BCI field. The *Journal of Neural Engineering* ranks first with 908 publications, accounting for 22.1% (908/4101) of the total among these top 10 journals, followed by *IEEE Transactions on Neural Systems and Rehabilitation Engineering* (577/4101 articles, 14.1%) and *Frontiers in Neuroscience* (489/4101, 11.9% articles). The remaining journals each published fewer than 400 articles. Among these 10 journals, 7 are classified as quartile 2 journals, while the other 3 are quartile 1 journals.

**Figure 4.**
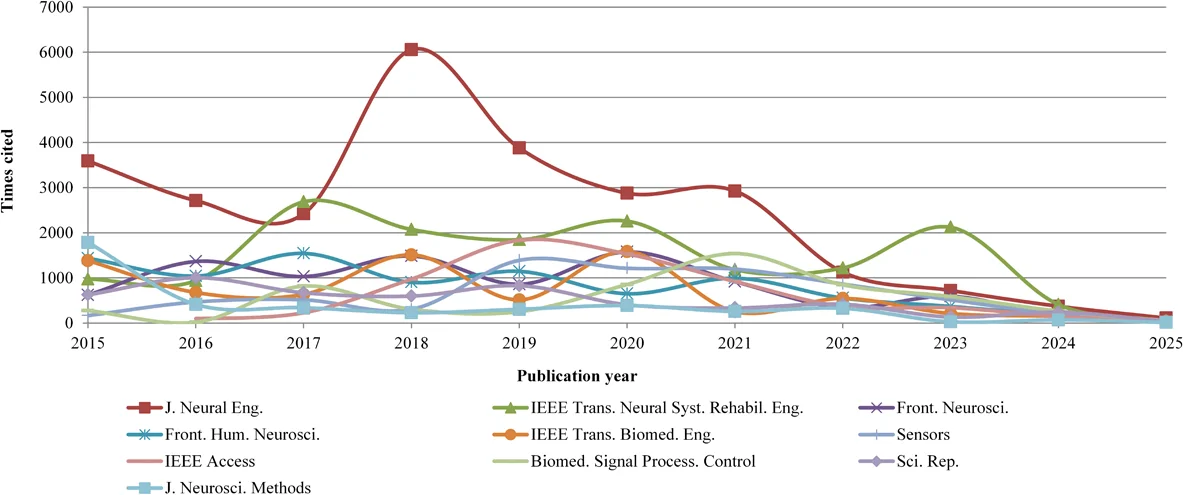
Annual citation trends of the top 10 most cited journals in the brain-computer interface (BCI) field.

Figure 4 illustrates the annual citation trends of the top 10 most cited journals in the BCI field. The *Journal of Neural Engineering* accumulated the highest total citations, peaking in 2018 before entering a sustained decline. This downward trajectory intensified after 2021 across almost all journals due to insufficient citation accumulation, with the notable exception of *IEEE Transactions on Neural Systems and Rehabilitation Engineering*, which demonstrated exceptional growth and reached a peak in 2023.

According to the WOS database, 157 HCPs and one Hot Paper (published within the past 2 years and ranked top 0.1% by citations for its category) were identified within our retrieved article dataset. The 3 journals with the highest number of HCPs are the *Journal of Neural Engineering* (12 papers), *IEEE Transactions on Biomedical Engineering* (8 papers), and *Nature Communications* (6 papers). The single Hot Paper, which was also identified as an HCP, was published in *Advanced Materials* in 2024 by researchers affiliated with a Chinese institution, presenting a flexible hydrogel electronics designed for smart BCI applications [22].

### Institution Analysis

Multimedia Appendix 2 demonstrates a comprehensive comparison of the top 10 influential institutions in the BCI field by article output, total citation frequency, and category normalized citation impact (CNCI). The CNCI of an article is calculated by dividing the actual count of citing items by the expected citation rate for articles with the same article type, year of publication, and subject area. The CNCI of a set of articles, for example, the collected articles of an individual, institution, country, or region, is the average of the CNCI values for all the articles in the set. The University of California System ranks first in total citation frequency, with 416 articles and 16,607 total citations. The institution with the largest number of BCI articles is the Chinese Academy of Sciences (504 articles and 14,975 citations), representing the only institution exceeding 500 articles. Stanford University achieves the highest CNCI among all institutions, yet produces fewer articles, indicating a quality-over-quantity strategy in BCI research. Among the top 10 institutions, 4 are based in the United States: the University of California System, Stanford University, Harvard University, and the Pennsylvania Commonwealth System of Higher Education (PCSHE). China and Switzerland are each represented by 2 institutions—the Chinese Academy of Sciences and Tsinghua University from China, and the Swiss Federal Institutes of Technology Domain and the École Polytechnique Fédérale de Lausanne from Switzerland.

Multimedia Appendix 3 presents a list of the primary applicants for patents related to BCI. Among the top 10 applicants, 9 are from China and one from South Korea. In terms of institutional types, 9 of the top 10 applicants are higher education institutions, and only one is an enterprise. Notably, Tianjin University ranks first on the list with over 50 granted patents. The focus areas of Tianjin University’s patent applications include “brain-computer interface systems based on motor imagery brain signal features,” “analysis of EEG signals based on deep or integrated learning,” and “brain-computer interfaces for controlling devices such as mice or wheelsets.”

### Country Analysis

Multimedia Appendix 4 presents a comprehensive comparison of the top 10 countries by article output, total citation frequency, and citation rate (% articles cited). Point radius in the visualization corresponds to citation rate, with larger radii indicating higher citation rates. It can be seen that both Germany and Switzerland exhibit exceptional citation rates, each exceeding 94%. The United States and China demonstrate distinct performance profiles that differentiate them from other leading countries. The United States ranks second in article output but attains the highest total citation frequency, indicating substantial aggregate research impact. Conversely, China leads in article output and ranks second in total citation frequency; however, it demonstrates the lowest citation rate among the top 10 countries, suggesting a productivity-impact disconnect.

Multimedia Appendix 5 shows that the United States has the largest number of international collaboration articles in the BCI field, whereas China exhibits the greatest number of domestic collaboration articles. Both nations occupy the top tier, substantially surpassing all other countries. Additionally, the number of international collaboration articles in Germany and the United Kingdom is much higher than that of domestic collaboration articles.

Table 1 ranks the top 10 countries by the number of BCI-related patents. China and the United States emerge as the primary technical contributors, with China leading by a substantial margin. The United States follows with 200+ patents, while South Korea, ranking third, has a patent volume approximately half that of the United States. The remaining seven countries each account for fewer than 100 patents.

The patent landscape across jurisdictions reflects distinct regional strategies of patent applicants. As shown in Table 2, patents originating from China are highly concentrated in its domestic market, with merely a small number deployed in the United States and South Korea. The United States conducts extensive patent layouts in the European Patent Office (EPO), China, South Korea, and Japan, showing remarkable characteristics of globalized patent deployment. For South Korea and Japan, their overseas patent filings are predominantly concentrated in the United States. By contrast, France has completed patent arrangements in the EPO and all the other 4 target countries.

**Table 1.** Top 10 countries by number of patents in the BCI^a^ field from 2015 to 2025.

Rank	Applicant country	Number of patents, n
1	China	960
2	United States	251
3	South Korea	127
4	Japan	29
5	France	28
6	Israel	14
7	India	14
8	Canada	13
9	Germany	13
10	Russia	13

a BCI: brain-computer interface.

**Table 2.** Patent disclosure layouts of the top 5 countries in the BCI^a^ field from 2015‐2025^b^.

Country	Publishing country or organization					
	EPO^c^	China	United States	South Korea	Japan	France
China	0	946	20	1	0	0
United States	23	13	217	12	18	0
South Korea	0	0	28	119	3	0
Japan	4	2	12	1	24	0
France	14	6	18	6	2	11

a BCI: brain-computer interface.

b Statistics based on nonmerged patent family counts.

c EPO: European Patent Office.

### Funding Agency Analysis

Bibliometric analysis revealed that the National Natural Science Foundation of China (NSFC), the United States Department of Health & Human Services, and the National Institutes of Health (NIH) ranked as the top 3 funding agencies in the BCI field by both article output and total citation frequency (Table 3). NSFC demonstrated marked dominance in article output, with a substantial lead over the second-ranked United States Department of Health & Human Services. Among the top 10 funding agencies, 4 are based in the United States, while 2 each are from China, Germany, and the European Union. The United States Department of Defense (DoD) and the National Key Research & Development Program of China (NKRDPC) have the highest percentages of articles in the top 1% by citations (% articles in top 1%), both exceeding 6%. Notably, among Chinese agencies, both NSFC and NKRDPC exhibit citation rates (% articles cited) below 90%.

**Table 3.** Statistics of the top 10 funding agencies of the brain-computer interface (BCI) field from 2015 to 2025.

Name	WOS^a^ articles	Times cited	% Articles Cited	% Articles in top 1%	HCPs^b^ count	HCPs cited by patents
National Natural Science Foundation of China	2081	46672	87.7 (1825)	3.9 (81)	44	9
United States Department of Health & Human Services	718	24735	93.9 (674)	3.2 (23)	21	7
National Institutes of Health- United States	710	24394	93.9 (667)	3.2 (23)	21	6
National Science Foundation	489	20569	95.1 (465)	5.3 (26)	19	6
United States Department of Defense	279	18791	97.1 (271)	6.5 (18)	13	6
German Research Foundation	233	10712	96.6 (225)	3.9 (9)	7	4
European Union	337	9803	94.4 (318)	2.4 (8)	8	3
National Key Research & Development Program of China	403	9648	88.1 (355)	7.4 (30)	18	1
Federal Ministry of Education & Research	170	9309	98.2 (167)	5.3 (9)	6	4
European Research Council	184	8030	94.0 (173)	4.4 (8)	9	4

a WOS: Web of Science.

b HCPs: highly cited papers.

Multimedia Appendix 6 illustrates the performance of the top 10 funding agencies across two normalized impact indicators: CNCI and impact relative to world (IRW). The DoD and the Federal Ministry of Education & Research (BMBF) demonstrated exceptional performance, with both agencies exceeding the threshold of 2.0 on both metrics. While the remaining agencies exhibited variable performance, the NSFC was the sole entity with an IRW value below 1.0, indicating below-world-average citation impact.

Analysis of the HCPs revealed that the top 10 funding agencies collectively supported 89 of the 157 HCPs. Among these, the NSFC contributed the most, funding 44 HCPs. Forward citation analysis showed that only 28 of these 89 HCPs were cited by patents, and merely 5 of them had citing patents from institutions ranked among the top 10 applicants (Multimedia Appendix 3). The funding agencies for these 5 articles were NSFC (3 articles) and the DoD (2 articles).

### Disruptive Trend Analysis

Following the methodology outlined in the Methods Section, we constructed citation networks and calculated CD5 values for articles and patents published up to 2020, ensuring complete 5-year observation windows. Annual averages were then derived (Figures 2 and 3, respectively). According to the calculation formula of the disruption index, the final value ranges from −1 (consolidating) to 1 (disruptive). Given that all CD5 values in Figures 2 and 3 are positive, both scientific research and technological innovation in the BCI field exhibited disruptive characteristics during the observation period, with new research and development practices continuously challenging and expanding existing knowledge boundaries.

Comparative analysis of Figures 2 and 3 reveals distinct patterns. Article CD5 values generally increased alongside publication volume, reaching a peak in 2020, the final year of complete observation. This trajectory suggests sustained growth in scientific disruptiveness. In contrast, patent CD5 values exhibited fluctuation within a bounded range rather than consistent growth. The divergence between rising scientific disruptiveness and the absence of a corresponding upward trend in patent disruptiveness raises the possibility that scientific paradigm shifts in BCI have not yet fully permeated the patent landscape, implying a potential translation lag. However, given the 5-year observation window and the likelihood of longer diffusion cycles, this interpretation remains speculative and requires validation through extended temporal analysis and direct science-to-patent citation tracking.

### Research Topics Analysis

#### Overview

**Figure 5.**
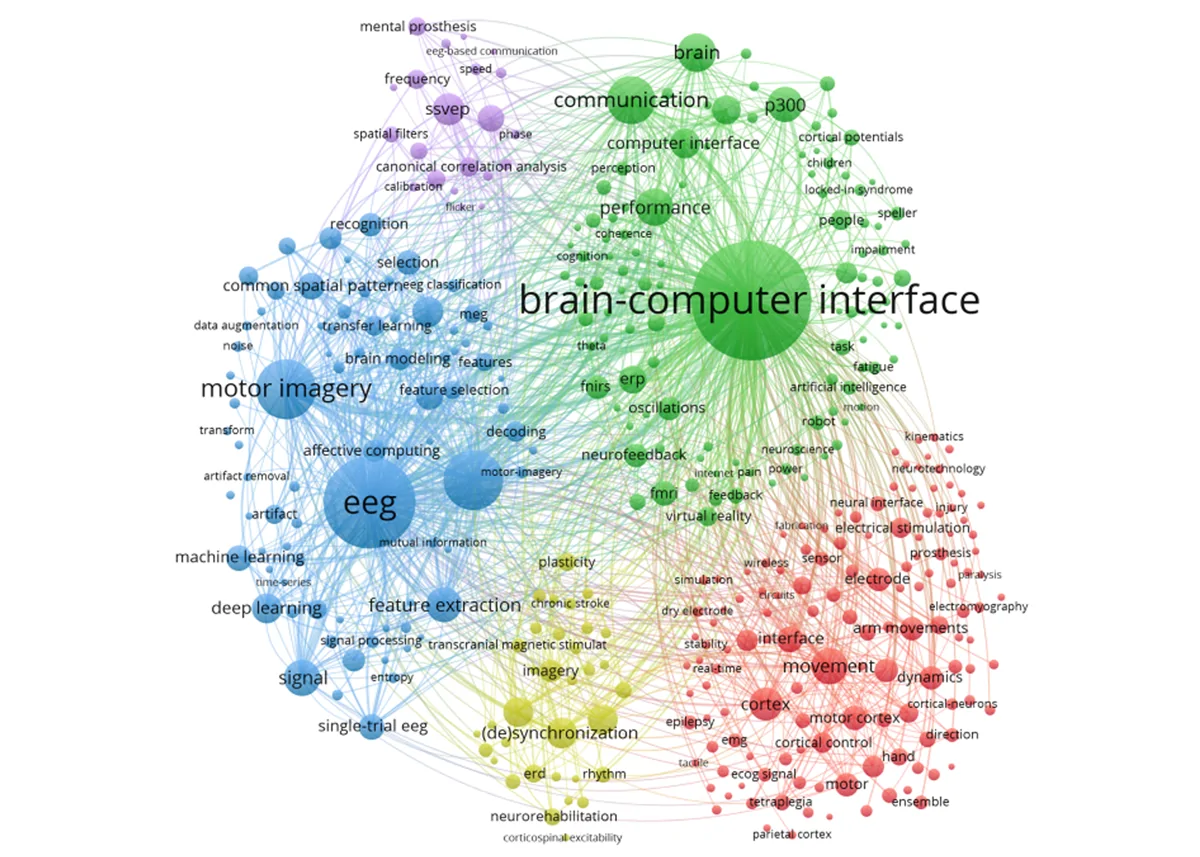
Keyword clusters of the brain-computer interface (BCI) field from 2015 to 2025.

Keywords with a cooccurrence frequency exceeding 30 were identified as high-frequency terms. This threshold was set to prioritize well-established and field-representative keywords, thereby revealing the core research structure of the field. Keywords were derived from author-supplied terms, with synonym normalization performed prior to analysis. For example, “BCI” and “brain-computer interface” were unified as “brain-computer interface”; “eeg” and “electroencephalogram” were unified as “eeg.” After synonym normalization, the high-frequency keywords were imported into VOSviewer for bibliometric mapping, where the link strength normalization method was set as association strength and clustering was configured with a minimum of 20 keywords per cluster, yielding five distinct clusters (Figure 5). These clusters centered on the following research topics: neural interfaces and motor control (red), brain-computer interface and cognitive neuroscience (green), electroencephalogram (EEG) signal processing and machine learning (blue), neurorehabilitation and stroke recovery (yellow), and steady-state visually evoked potentials (SSVEP) & system optimization (purple).

#### Cluster 1: Neural Interfaces and Motor Control

This cluster (represented in red) comprised 133 keywords, with prominent nodes including movement, cortex, motor cortex, neurons, neural interface, spinal-cord-injury, and assistive technology. The thematic focus centered on motor control mechanisms and the application of neural interface technologies, particularly the decoding and modulation of neural activity in the motor cortex. Key methodologies involved signal acquisition via electrodes or microelectrode arrays to record neuronal activity, enabling precise control of arm and hand movements or functional recovery in spinal-cord-injury patients. Keywords such as brain-machine interface and neuroprosthesis highlighted applications in assistive technology and tetraplegia. Further investigations extended to neuromodulation therapies (eg, deep brain stimulation) for neurological disorders like Parkinson disease.

#### Cluster 2: BCI and Cognitive Neuroscience

This cluster (represented in green) encompasses 111 keywords, including prominent nodes such as BCI, communication, performance, P300, functional magnetic resonance imaging (fMRI), and amyotrophic lateral sclerosis (ALS), as well as smaller yet significant nodes like AI, virtual reality, human-computer interaction, augmented reality, and neurophysiology. Thematic analysis reveals that this cluster centers on BCI, emphasizing their applications in communication and disease intervention.

High-frequency keywords such as P300 and Event-Related Potential (ERP) reflect a concentrated focus on EEG signal decoding, while functional near-infrared spectroscopy (fNIRS) and fMRI indicate the integration of multimodal neuroimaging techniques. Key research objectives within this direction encompass restoring interaction capabilities for patients with ALS and locked-in syndrome, as well as exploring the synergy between virtual reality and neurofeedback technologies. Additionally, cognitive mechanisms such as working memory and attention are incorporated into studies aimed at optimizing BCI performance.

#### Cluster 3: EEG Signal Processing and Machine Learning

This cluster (represented in blue) encompasses 80 keywords, with central nodes including EEG, classification algorithms, motor imagery, deep learning, convolutional neural network (CNN), neural networks, and machine learning, complemented by specialized terms such as data augmentation, genetic algorithm, principal component analysis (PCA), and ensemble learning. The thematic focus centers on algorithmic processing and pattern recognition of EEG signals.

Key research areas include motor imagery and single-trial EEG analysis, with widely used methodologies such as CNN and common spatial pattern (CSP). Keywords such as artifact removal and feature extraction highlight the critical role of signal preprocessing, while emerging analytical approaches (eg, Riemannian geometry and wavelet transform) reflect ongoing methodological advancements. The research scope extends to translational domains such as affective computing and seizure detection, emphasizing the interdisciplinary integration of machine learning in neural engineering.

#### Cluster 4: Neurorehabilitation and Stroke Recovery

This cluster (represented in yellow) consists of 34 keywords, including significant nodes such as stroke, rehabilitation, plasticity, neurorehabilitation, and transcranial magnetic stimulation, as well as smaller nodes like sensorimotor rhythms, chronic stroke, direct-current stimulation, and induced movement therapy. The thematic focus of this cluster is centered on motor recovery and neuroplasticity mechanisms following a stroke. Key therapeutic approaches encompass functional electrical stimulation and exoskeleton-assisted therapy, with rehabilitation efficacy evaluated through EEG-derived features such as event-related desynchronization (ERD) and mu-rhythm modulation. Notably, terms such as chronic stroke and upper-limb highlight specific patient subpopulations, while neuromodulation techniques (eg, transcranial magnetic stimulation [TMS] and direct-current stimulation) are used to regulate cortical excitability. The prevailing research paradigm integrates motor execution and motor imagery to promote functional reorganization of neural circuits.

#### Cluster 5: SSVEP and System Optimization

This cluster (represented in purple) consists of 21 keywords, with central nodes including SSVEP, potentials, frequency, canonical correlation analysis (CCA), and evoked potentials, complemented by specialized terms such as hybrid BCI and enhancing detection. Thematic analysis indicates that this cluster primarily revolves around SSVEPs, with a focus on high-frequency visual stimuli (eg, flicker frequency) to elicit neural responses. CCA serves as the primary decoding algorithm, used to enhance the signal-to-noise ratio (SNR). Keywords such as hybrid BCI and cursor control highlight applications in interactive systems, while topography and phase reflect explorations into spatial and temporal signal characteristics. The research objectives within this direction include developing rapid calibration methods and detection enhancement techniques to optimize real-time BCI systems.

#### Research Topics Evolutionary Analysis

**Figure 6.**
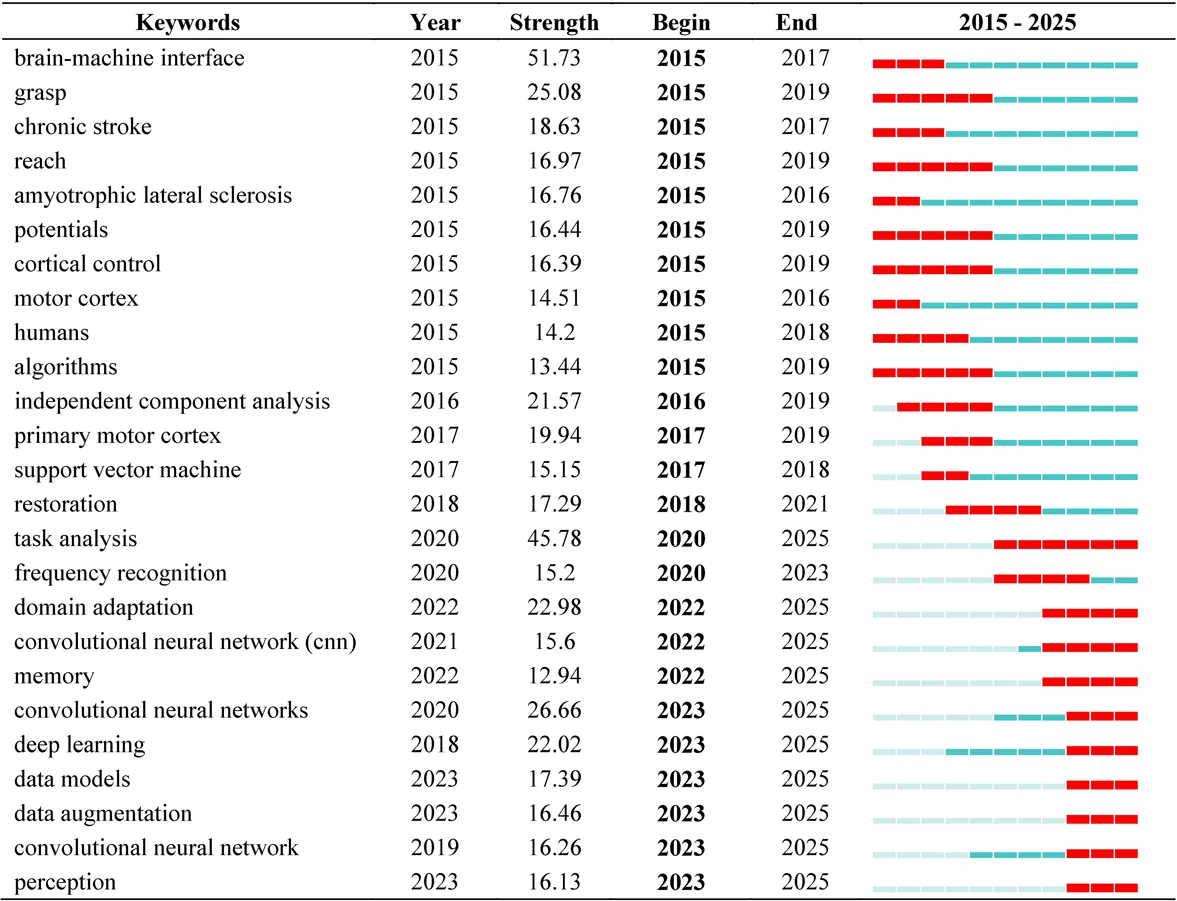
Top 25 strongest citation burst keywords in the brain-computer interface (BCI) field from 2015 to 2025. BCI: brain-computer interface.

Based on the burst detection function of CiteSpace, a longitudinal analysis of burst keywords in the BCI field reveals that research topics over the past decade can be broadly categorized into 3 distinct stages (Figure 6).

- Fundamental neural mechanism exploration (2015-2017): "Brain-machine interface" ranked first with burst strength of 51.73, cooccurring with “motor cortex” and “amyotrophic lateral sclerosis,” indicating a research focus on motor control mechanisms and neurodegenerative disease applications dominated by classical physiological paradigms.
- Algorithmic transition (2018-2021): The successive emergence of “restoration” (17.29) and “deep learning” (22.02) marked a shift from conventional signal processing to AI algorithms, with machine learning techniques such as “support vector machine” gaining traction.
- Deep learning dominance (2022-2025): Terms including “domain adaptation,” “convolutional neural networks,” and “data augmentation” demonstrated concentrated bursts during 2022-2023, demonstrating that CNN architectures and data-driven approaches have become the current research frontier. The sustained burst of “task analysis” (2020-2025) further confirms the deepening expansion of application scenarios.

### International Patent Classification Analysis

The International Patent Classification (IPC) codes, established by the World Intellectual Property Organization (WIPO), served as the analytical framework for mapping patent landscapes. BCI-related patents were categorized according to IPC classifications, and the top 10 technical topics ranked by the number of patents were selected. As shown in Table 4, key technical areas include input devices for transforming data into a computer-processable format or output devices for transferring data from a processor (G06F3), medical diagnostic measurements (A61B5), pattern recognition methods or devices (G06F18 and G06K9), and biologically modeled computer systems (G06N3).

**Table 4.** Top 10 IPC^a^ numbers of the BCI^b^ field from 2015 to 2025.

IPC (main group)	Number of patents, n
G06F3 (Input devices for transforming data to a form capable of being processed by a computer; output devices for transferring data from a processor to output equipment, eg, interface devices)	737
A61B5 (Measuring for diagnostic purposes; identification of humans)	734
G06F18 (Pattern recognition)	285
G06N3 (Computer systems based on specific computational models)	238
G06K9 (Methods or devices for recognizing patterns)	105
A61N1 (Electrotherapy; apparatus therefor)	102
G06N20 (Machine learning)	62
A61F2 (Filters to be implanted into blood vessels; prostheses, ie, artificial substitutes or replacements for parts of the human body; appliances for connecting prostheses to the human body; devices for providing openings in body tissues or for preventing collapse of tubular structures in the human body, eg, stents)	52
G06F17 (The specialized digital computing or data processing equipment or methods for specific functions)	44
B25J9 (Programmed manipulators)	42

a IPC: international patent classification.

b BCI: brain-computer interface.

### Dual Citation Analysis

**Figure 7.**
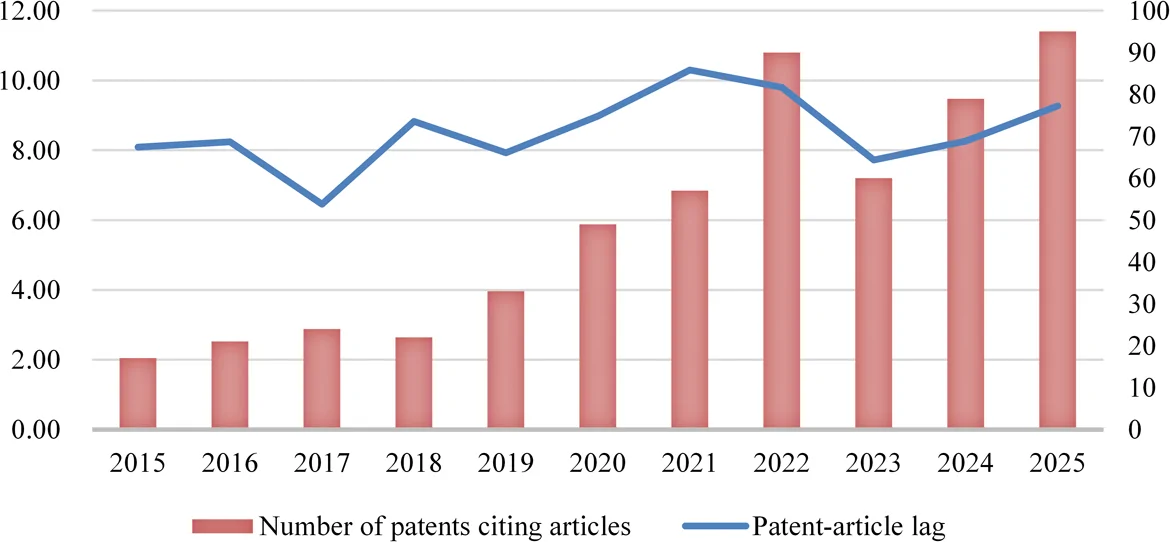
Trends of the number of patents citing articles and patent-article lags.

Backward citation analysis revealed that 547 of the 1551 granted patents (35.3%) collectively cited 2726 articles; however, 397 of these 547 patents (72.6%) cited only one article each, indicating a pronounced long-tail distribution. The mean time lag between the patent grant year and the publication year of the cited articles was 8.8 years. Further analysis of the annual citation time lags (Figure 7) revealed that the number of patents citing articles increased in tandem with the total number of granted patents; however, the year lag between the patent grant year and the publication year of the cited articles fluctuated within the range of 6.5‐10.3, showing no discernible unidirectional trend.

Forward citation analysis revealed that 50 of the 157 HCPs were cited by patents after publication. Simultaneous examination of the article publication institutions and patent application institutions revealed that for only 10 of the 157 HCPs, the publishing institutions were also the applicants of the patents that cited them. These 10 institutions were: Ecole Polytechnique Federale de Lausanne, Guangdong University of Technology, Harvard University, Huazhong University of Science & Technology, Imperial College London, King Abdullah University of Science & Technology, Massachusetts Institute of Technology, Neuralink Corp, Shenzhen University, and University of Pittsburgh. Similar to the top 10 applicants of the granted patents (Multimedia Appendix 3), 9 of these 10 institutions were higher education institutions, with Neuralink Corp being the only nonacademic institution.

## Discussion

### Principal Findings

The sustained growth in both BCI articles and patents over the past decade reflects the development of this interdisciplinary field, bridging neuroscience, engineering, and computer science. The parallel upward trajectories of scientific output and technological protection suggest a robust innovation ecosystem where fundamental research advances are translated into proprietary technologies. Promoters of scientific research and technological innovation at different levels, such as journals, institutions, countries, and funding agencies, have collectively facilitated this expansion; however, their respective contributions and impacts are different.

The concentration of BCI articles in specialized engineering and neuroscience journals reflects the field’s strong methodological orientation. The *Journal of Neural Engineering*, despite its leading productivity, exhibited sharply declining citations after 2018. In contrast, *IEEE Transactions of Neural Systems and Rehabilitation Engineering* demonstrated exceptional growth, peaking in 2023 while others declined due to insufficient citation accumulation. This divergence suggests that the latter’s publications have attracted disproportionate disciplinary attention, potentially reflecting timely responses to emerging methodological demands. The sole Hot Paper, published in *Advanced Materials* by Chinese researchers, underscores the emerging convergence between materials science and BCI development, signaling a potential shift toward translational applications requiring advanced hardware solutions.

The institutional landscape reveals distinct strategies in BCI research performance. Stanford University achieves the highest CNCI despite modest article output, indicating a quality-over-quantity approach that prioritizes foundational impact. Conversely, both the Chinese Academy of Sciences and the University of California System have gained substantial influence through scaled production: the former leads in article output, while the latter generates the greatest total citation frequency. This divergence suggests that elite institutions can achieve research leadership through alternative pathways, selective high-impact contributions or comprehensive engagement, reflecting differing mission orientations and resource allocation strategies within the field.

The divergent performance profiles of the United States and China illustrate contrasting national strategies in BCI research. The United States achieves the highest aggregate citation impact despite ranking second in article output, coupled with the largest number of international collaborations articles—suggesting a network-centric approach that leverages global partnerships to amplify research influence. China, while dominating in article output and domestic collaboration intensity, exhibits the lowest citation rate among leading nations, indicating a potential quantity-quality trade-off. This productivity-impact disconnect may reflect differing evaluation incentives or developmental stages of research ecosystems. The substantial gap between these 2 nations and all others underscores their central positioning in the global BCI research architecture, yet their divergent pathways imply distinct trajectories for shaping the field’s future directions.

Similar to the analysis results of the articles, China and the United States form a distinct top tier in global BCI patenting, substantially exceeding all other nations. However, their internationalization strategies diverge markedly. While both prioritize domestic patent filings, the United States demonstrates systematic expansion across EPO and foreign national markets—indicating a deliberate strategy to secure global market access. In contrast, China’s limited extraterritorial presence, despite its leadership of patent output, suggests an orientation toward domestic exploitation rather than international competition. Meanwhile, patent analysis at the institutional level reveals remarkable concentration in higher education institutions, with Tianjin University ranked first, reflecting systemic incentives for academic engagement in technology transfer. However, the near-absence of nonacademic applicants in the top tier indicates a university-centric commercialization ecosystem, potentially constraining rapid market translation compared to industry-led innovation models.

The funding landscape reveals distinct agency approaches to BCI research investment. China’s NSFC operates as a high-volume engine, substantially outpacing all other agencies in article output, yet exhibits below-world-average citation impact (IRW<1.0) and a citation rate below 90%. This volume-impact disconnect suggests that scale-oriented funding strategies may not automatically translate into commensurate research influence. Conversely, the DoD and Germany’s BMBF achieve exceptional normalized impact (CNCI and IRW both exceeding 2.0), indicating highly selective, high-return investment approaches. Intranational comparison reveals that China’s NKRDPC, tasked with strategic technology development, achieves the highest percentage of articles in the top 1% by citations (7.4%) among all funding agencies, outperforming NSFC (3.9%) in impact concentration. This intranational divergence (NKRDPC vs NSFC) suggests that mission-oriented funding with clear technological targets may yield higher per-unit impact compared to investigator-driven schemes, offering a pattern with implications for science policy design globally. Additionally, forward citation analysis of HCPs revealed that, although mainstream funding agencies made substantial contributions to the output of HCPs, the overall efficiency of translating these high-impact research achievements into patents remained low, with only a limited number of such outputs attracting the attention of top-ranking patent applicants. This indicates that the linkage mechanism between research funding orientation and technology transfer still warrants further strengthening.

Regarding the disruptive trends in the BCI field, although the observation period of this study is limited, preliminary comparisons can still be drawn with existing research. Park et al [21] analyzed 25 million articles (1945‐2010) and 3.9 million patents (1976‐2010) using the Disruptive Index and demonstrated a universal decline in disruptiveness across science and technology. The sustained growth in BCI scientific disruptiveness through 2020 suggests that this interdisciplinary field continues to generate novel conceptual frameworks that challenge existing knowledge boundaries. However, the fluctuating and directionless trajectory of patent disruptiveness indicates that recent scientific paradigm shifts have not yet fully permeated the patent landscape. This asynchrony, rising scientific disruptiveness alongside the absence of an upward trend in patent disruptiveness, is consistent with potential translational friction between academic advances and technological outputs, warranting continued monitoring as the field evolves.

The thematic evolution of BCI research demonstrates a clear trajectory from neural mechanism exploration through algorithmic transition to application deepening. Initially centered on classical physiological paradigms investigating motor control and neurodegenerative diseases, the field underwent a fundamental shift toward AI-driven approaches, with deep learning architectures now dominating both research and technological development. This scientific progression aligns closely with the patent landscape, where pattern recognition methods (G06F18 and G06K9) and biologically modeled computing systems (G06N3) feature prominently alongside medical diagnostic measurements (A61B5) and human-computer interaction technologies (G06F3). The emergence of pattern recognition methods as core technical categories particularly underscores the critical role of algorithmic innovation in enabling reliable neural decoding. The parallel emphasis on algorithmic sophistication in both research bursts and patent classifications suggests substantial potential for advancing deep learning applications within the BCI field.

Due to variations in developmental stages and technological application environments, the time lag for knowledge transfer from articles to patents differs across fields [23]. Emerging fields generally exhibit shorter translation timelines, typically estimated at 3‐4 years [24,25]. This study found that the time lag between the patent grant year and the publication year of the cited articles in the BCI field was 8.8 years. Combined with the percentages obtained from the bidirectional citation analysis—such as 1004 of 1551 patents (64.7%) citing no articles, 107 of 157 HCPs (68.2%) receiving no patent citations, and the limited number and type of institutions achieving internal translation (concentrated within higher education institutions)—these findings collectively suggest that knowledge translation from scientific research to technological innovation in this field faces certain barriers. Our findings are partially consistent with prior research on human-computer interaction (HCI), a closely related and broader field, where the time lag between the patent grant date and the publication date of cited papers was reported as 10.5 years, leading to the conclusion that research and practice in the HCI domain may not be efficiently connected [26].

### Conclusions

This study provides a comprehensive scientometric characterization of the BCI field through dual analysis of articles and patents. The findings reveal a rapidly expanding yet strategically differentiated global landscape: the United States and China constitute a distinct top tier in both scientific output and technological innovation, China leads in article output and patent quantity with intensive domestic collaboration, while the United States achieves the highest citation impact and most extensive international patent portfolios through globalized research networks. Institutional and funding-level analyses further demonstrate that research leadership can be achieved through alternative strategies, whether selective quality-focused investment or comprehensive volume-driven engagement, with mission-oriented funding demonstrating superior per-unit impact compared to investigator-driven schemes. However, the linkage mechanism between research funding orientation and technology transfer still warrants further strengthening.

A critical finding emerges from the divergent disruptiveness trajectories: while BCI scientific research maintains active disruptive potential, technological development exhibits no synchronous upward trajectory. This asynchrony, combined with prolonged knowledge transfer lag, a high proportion of HCPs receiving no patent citations and patents citing no prior articles, and an academic-dominated patent landscape, collectively suggests certain barriers to knowledge translation in this field. The field’s thematic evolution from neural mechanisms through AI integration to application-specific deployment traces a discernible disciplinary trajectory; however, bridging the gap between scientific capability and technological implementation requires strengthened mechanisms to facilitate translation from scientific advances into technical innovations.

### Strengths and Limitations

This study simultaneously selected articles and patents in the BCI field for synchronous bibliometric and visualized analysis, which can obtain relatively objective and comprehensive analysis results, and thereby draw reliable conclusions. Moreover, the application of the Disruptive Index can achieve nuanced characterization of the development trends of scientific research and technological innovation in the BCI field. However, limitations also exist in our study. First, WOS Core Collection was selected as the only data source for articles, so publications indexed exclusively in other databases such as PubMed or Scopus might be left out. Second, keyword inclusion thresholds for topic analysis resulted in limited coverage of emerging domain concerns, such as neural data ownership, ethics, and privacy security. Third, the CD5 index requires a 5-year forward citation window, restricting this analysis to publications through 2020. As a result, post-2020 disruptive trends, including the impact of the AI boom on BCI, fall outside the scope of this study. In the future, we will conduct longitudinal tracking of the BCI field based on the analytical framework established in this study.

## Supplementary material

10.2196/95902 Multimedia Appendix 1 Top 10 journals by number of brain-computer interface–related articles from 2015 to 2025.

10.2196/95902 Multimedia Appendix 2 Top 10 most influential institutions in the brain-computer interface field from 2015 to 2025.

10.2196/95902 Multimedia Appendix 3 Top 10 institutions by number of granted patents in the brain-computer interface field from 2015 to 2025.

10.2196/95902 Multimedia Appendix 4 Top 10 most influential countries in the brain-computer interface field from 2015 to 2025.

10.2196/95902 Multimedia Appendix 5 Top 10 countries by number of international and domestic collaboration articles in the brain-computer interface field from 2015 to 2025.

10.2196/95902 Multimedia Appendix 6 Impact of the top 10 funding agencies of the brain-computer interface field from 2015 to 2025.

## Abbreviations

ALS: amyotrophic lateral sclerosis
BCI: brain-computer interface
BMBF: Federal Ministry of Education & Research
CCA: canonical correlation analysis
CD5: five-year disruptive index
CNCI: category normalized citation impact
CNN: convolutional neural network
CSP: common spatial pattern
DoD: United States Department of Defense
EEG: electroencephalogram
EPO: European Patent Office
ERD: event-related desynchronization
ERP: event-related potential
fMRI: functional magnetic resonance imaging
fNIRS: functional near-infrared spectroscopy
HCI: human-computer interaction
HCP: highly cited papers
IPC: International Patent Classification
IRW: impact relative to world
NIH: National Institutes of Health
NKRDPC: National Key Research & Development Program of China
NSFC: National Natural Science Foundation of China
PCA: principal component analysis
PCSHE: Pennsylvania Commonwealth System of Higher Education
SNR: signal-to-noise ratio
SSVEP: steady-state visually evoked potential
TMS: transcranial magnetic stimulation
WIPO: World Intellectual Property Organization
WOS: Web of Science
